# Serum Hepcidin-25 and Risk of Mortality in Patients on Peritoneal Dialysis

**DOI:** 10.3389/fmed.2021.684548

**Published:** 2021-06-17

**Authors:** Zhong Zhong, Dan Luo, Ning Luo, Bin Li, Dongying Fu, Li Fan, Zhijian Li, Wei Chen, Haiping Mao

**Affiliations:** ^1^Department of Nephrology, The First Affiliated Hospital, Sun Yat-sen University, Guangzhou, China; ^2^Key Laboratory of Nephrology, National Health Commission and Guangdong Province, Guangzhou, China; ^3^Clinical Trials Unit, The First Affiliated Hospital, Sun Yat-sen University, Guangzhou, China

**Keywords:** peritoneal dialysis, serum hepcidin-25, mortality, cohort study, prognostic factor

## Abstract

**Background:** Increased serum hepcidin-25 level is associated with excess mortality in hemodialysis patients. However, there is a dearth of published information about its predictive effect for survival in patients on peritoneal dialysis (PD). The purpose of this study is to evaluate the association of serum hepcidin-25 with the risk of mortality in PD patients.

**Methods:** Serum hepcidin-25 level was measured using an enzyme-linked immunosorbent assay in a prospective cohort study of PD patients with stored serum samples at baseline. Multivariate linear regression model was used to determine clinical characteristics associated with serum hepcidin-25 concentration. We evaluated the relationship between serum hepcidin-25 and all-cause mortality using a Cox proportional hazards model and the relationship between hepcidin-25 and cardiovascular (CV) and infection-related deaths using competing-risks regression models.

**Results:** In total, 513 PD patients were included in this study. The median serum hepcidin-25 level was 40.9 (17.9–85.9) ng/mL. Body mass index and serum ferritin were positively correlated with serum hepcidin-25 levels. During a median follow-up period of 64.1 months, 122 (24%) patients died, including 61 (50%) CV deaths and 32 (26%) infection-related deaths. In multivariable analysis, patients with the highest tertile of serum hepcidin-25 had a greater risk of all-cause [adjusted hazard ratio (aHR) 1.85, 95% confidence interval (95%CI), 1.14 to 3.00, *P* = 0.013] and infection-related mortality (adjusted subdistribution hazard ratio [aSHR], 2.61; 95%CI, 1.01 to 6.76, *P* = 0.049) when compared with those in the second tertile. However, no significant relationship was observed between serum hepcidin-25 and CV mortality.

**Conclusions:** Higher baseline serum hepcidin-25 level was associated with increased risk for all-cause and infection-related mortality in PD patients.

## Introduction

Anemia, a common complication of chronic kidney disease (CKD), is associated with significant morbidity and mortality (1). The primary cause of anemia in CKD is the reduced production of erythropoietin (EPO) (2, 3), however, approximately 5–43% of patients exhibit hyporesponsiveness to erythropoiesis-stimulating agents (ESAs) despite adequate dosing (4, 5). Iron deficiency, both absolute (AID) and functional (FID), has been recognized as an independent risk factor for ESA hyporesponsiveness (6). Importantly, PD patients have a high incidence of iron deficiency (7), and our recent study also found that iron deficiency was present in 77.5% of PD patients (8). AID is a deficit in total body iron and can be treated by iron supplementation, whereas iron sequestration in the reticuloendothelial system is the hallmark of FID (9). The causes of FID are multifactorial, but it can be present in many acute and chronic inflammation states and tightly related to cytokine induced hepcidin synthesis (9).

Hepcidin, a hepatogenic peptide hormone, is a major regulator of systemic iron homeostasis. Hepcidin-25, the bioactive form of hepcidin, interacts with the cellular iron exporter ferroportin and induces internalization and degradation of ferroportin, thereby resulting in decreased dietary iron absorption and increased intracellular iron stores in macrophages and hepatocytes (10, 11). Besides, hepcidin may also be involved in host defense by sequestering iron from microbes (12). Given that hepcidin is cleared by the kidney, its serum levels are increased in renal failure patients with anemia (13, 14) and associated with iron-restricted erythropoiesis and resistance to recombinant human erythropoietin treatment (14). In addition, a significantly positive correlation was found between carotid intima-media thickness and serum hepcidin-25 levels in hemodialysis (HD) patients, and serum hepcidin-25 levels were markedly lower in survivors than in patients who died of cardiovascular disease (CVD) (15). Niikura et al. reported that PD patients have higher levels of serum hepcidin compared to non-dialysis CKD and HD patients (16). Further, increased hepcidin levels were associated with atherosclerosis (17) and arterial stiffness (18) in PD patients. However, the relationship between serum hepcidin and mortality in PD patients remains unclear.

In the current study, we aimed to determine whether serum hepcidin-25 levels can predict the prognosis of PD patients through a cohort study.

## Materials and Methods

### Study Design and Study Population

From January 2009 to December 2013, we performed an observational cohort study of patients who underwent continuous ambulatory peritoneal dialysis (CAPD) at our PD center. Eligible participants were 18 years or older, treated with CAPD for more than 3 months and had stored serum samples at baseline. Patients were excluded if they had been previously receiving HD or renal transplantation, had a history of peritonitis at baseline, or baseline data missing on serum ferritin and transferrin saturation (TSAT). This study was conducted according to the Helsinki Declaration, and the study protocol was approved by the Ethics Committee of The First Affiliated Hospital of Sun Yat-sen University. All participants gave their written informed consent.

### Clinical Data Collection

Baseline demographic and clinical data were collected within the week preceding PD catheter implantation. Clinical data included age, gender, body mass index (BMI), underlying renal disease, comorbidities, and anemia medication. Comorbidities were assessed using the Charlson Comorbidity Index (CCI) (19). Baseline laboratory information at the first 1–3 months after the initiation of CAPD, including serum hepcidin-25, hemoglobin, serum albumin, high-sensitivity C-reactive protein (hs-CRP), albumin-corrected calcium, phosphate, uric acid, total cholesterol, triglyceride, serum iron, total iron binding capacity (TIBC), TSAT and ferritin, were recorded. Dialysis adequacy was assessed by weekly Kt/V urea using data from 24-h dialysate and urine collections. Residual renal function (RRF) was determined as the average values of urinary urea and creatinine clearance adjusted for body surface area. During follow-up, all data elements obtained from medical records were reviewed for accuracy by nephrologists and nurses in our PD center.

### Measurement of Hepcidin-25

Serum samples were stored at −80°C until assayed. Serum hepcidin-25 levels were measured in duplicate using a commercially available enzyme immunoassay kit (S-1337; Bachem/Peninsula Laboratories, San Carlos, CA, USA) according to the manufacturer's instructions.

### Outcomes

The primary outcome was all-cause mortality, and the secondary outcome included infection-related and CV mortality. CV death is mainly caused by congestive heart failure, cardiac arrhythmia, cardiac arrest, acute myocardial infarction, intracranial hemorrhage, cerebral infarction, and peripheral vascular disease (20). To determine the cause of death for each case, three nephrologists at our PD center thoroughly reviewed medical records and/or directly communicated with the referring physician. For mortality analyses, patients were censored at the time of transfer to HD treatment or other peritoneal dialysis centers, kidney transplantation, lose to follow-up or the end of the study period (December 31, 2018).

### Statistical Analysis

The patients were categorized into three groups according to the tertiles of baseline serum hepcidin-25 levels as follows: tertile 1 ( ≤ 24.97 ng/mL), tertile 2 (24.97–64.23 ng/mL), and tertile 3 (≥64.23 ng/mL). All continuous variables were presented as mean ± standard deviation for normally distributed data and the median (interquartile range [IQR], 25th and 75th percentiles) for non-parametric data. For multiple group comparisons, one-way ANOVA or Kruskal-Wallis tests were used to analyze continuous data, as appropriate. Categorical variables were summarized as frequency (%), and chi-square tests were applied to compare categorical data.

To identify the clinical parameters independently associated with serum hepcidin-25 levels, we used natural log-transformed values to approximate normal data distribution and performed multivariable linear regression analyses using a stepwise conditional method. The cumulative survival was calculated by Kaplan-Meier method and log-rank test was used to compare the survival curves. We conducted a restricted cubic spline model with five knots to evaluate the shape of the relationship between serum hepcidin-25 levels and all-cause mortality. The association of baseline serum hepcidin-25 with all-cause mortality was assessed with Cox proportional hazard regression models. Competing-risks regression models were used to examine the associations between serum hepcidin-25 and CV and infection-related mortality. When CV or infection-related deaths were modeled, other causes of death were treated as competing events. Unadjusted associations were first examined followed by adjustments for age, gender, and BMI, uric acid, corrected calcium, phosphorus, albumin, comorbidity score, hemoglobin, RRF, total Kt/V, hs-CRP, serum ferritin and treatment of ESAs and/or iron to examine whether hepcidin-25 was independently associated with mortality. The results were presented as hazard ratios (HRs) or subdistribution HR (SHR) with 95% confidence intervals (CIs). The proportional hazards assumption was verified by examination of scaled Schoenfeld residual plots. All statistical analyses were performed by using SPSS software, version 22.0 (IBM SPSS, Chicago, IL, USA) and R statistical software (version 3.6.1). A two-sided *P*-value of < 0.05 was considered statistically significant.

## Results

### Baseline Characteristics of the Entire Cohort

**Figure 1:**
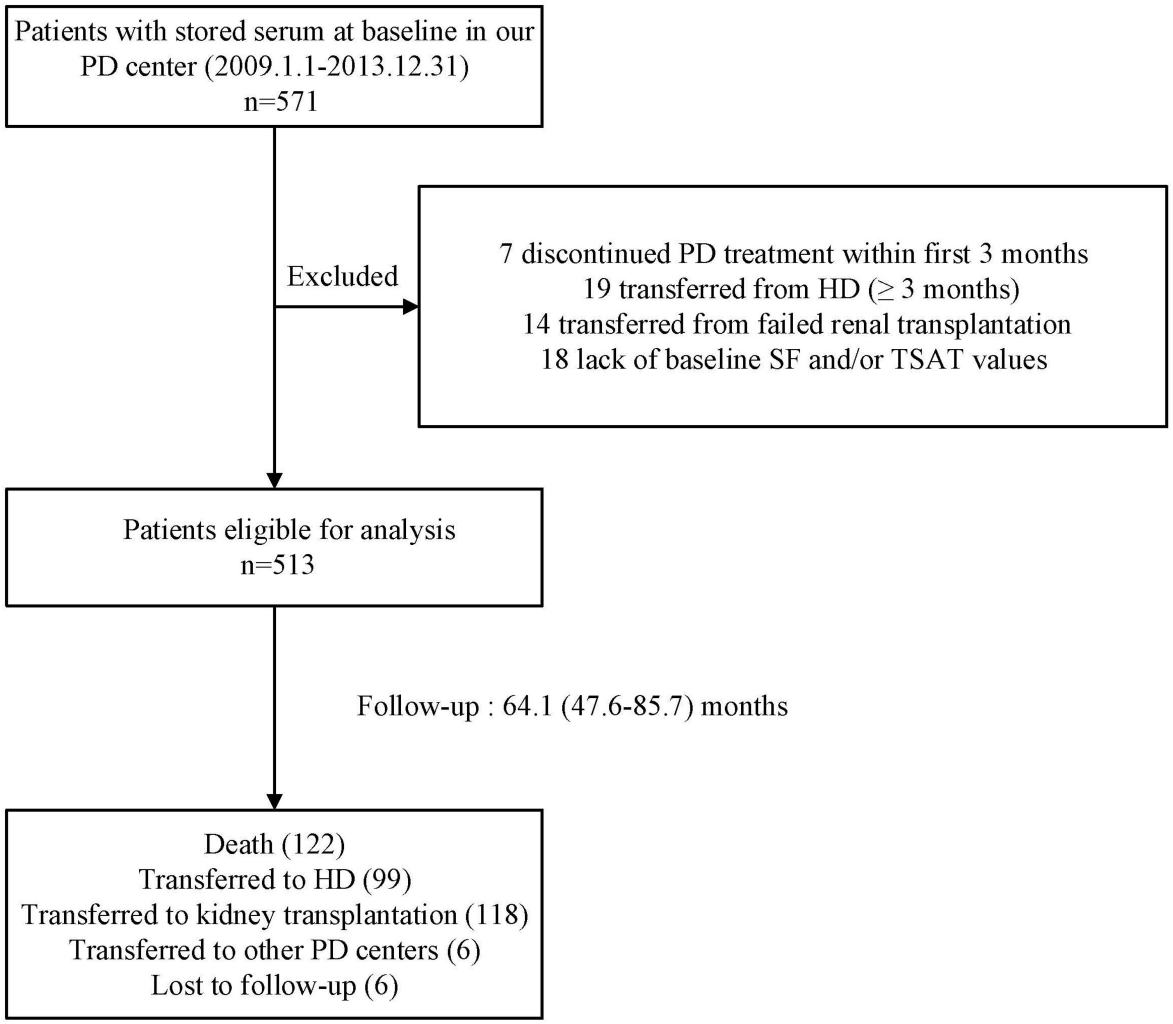
Flow chart of the participants in the study cohort. PD, peritoneal dialysis; HD, hemodialysis; SF, serum ferritin; TSAT, transferrin saturation.

**Figure 2:**
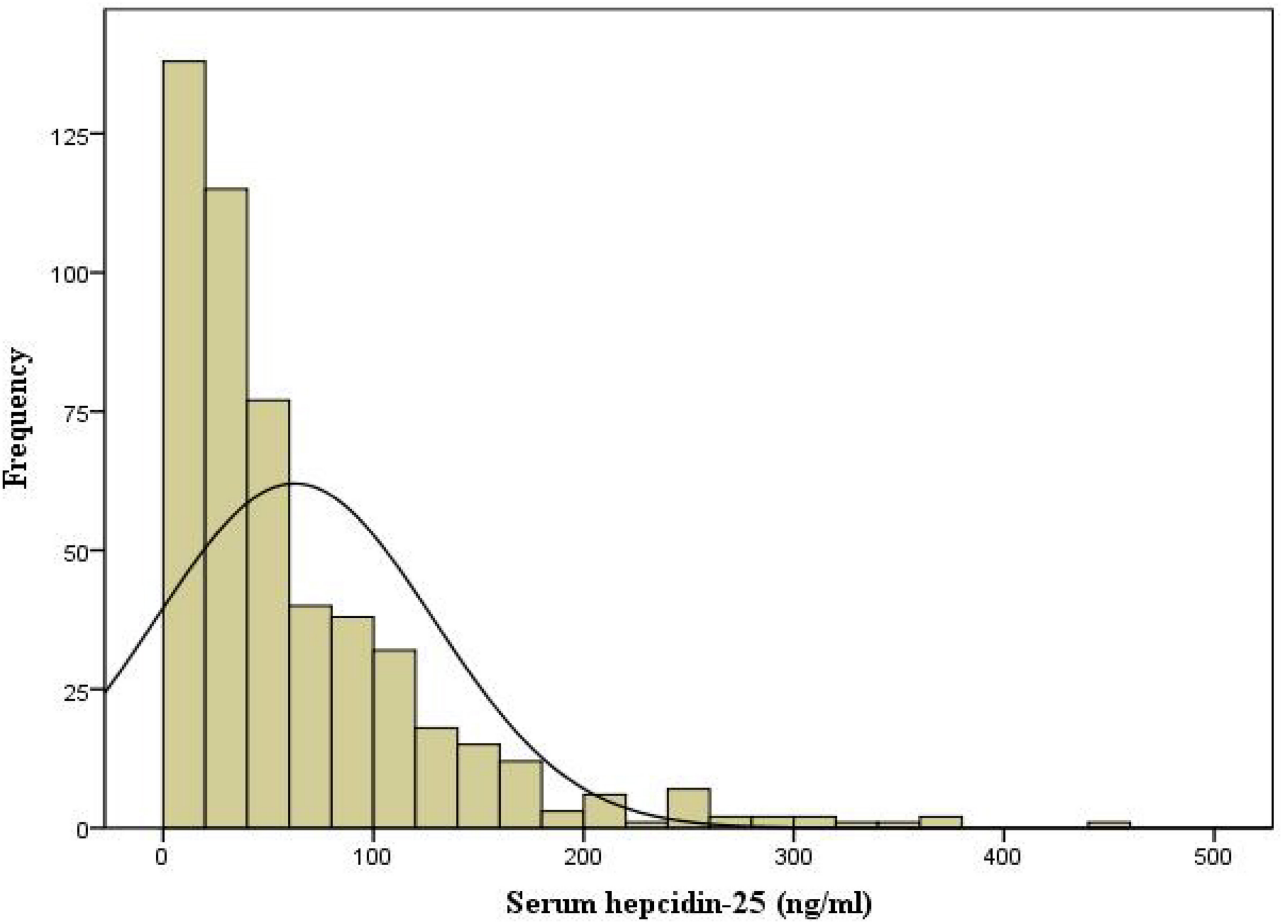
Distribution of serum hepcidin-25 in our cohort.

The flow chart of patient for the study is shown in Figure 1. A total of 513 PD patients were enrolled. The mean age was 46 ± 14 years; 309 (60%) of patients were males, 122 (24%) had diabetes, and 194 (38%) had a history of CVD. Figure 2 reveals a positively skewed distribution of baseline serum hepcidin-25 level among the overall study population. The median serum hepcidin-25 level was 40.9 ng/mL (IQR, 17.9–85.9 ng/mL), and male patients tend to had a higher median serum hepcidin-25 level than female patients (42.97 [21.08–95.29] vs. 39.27 [13.40–75.54] ng/mL, respectively; *P* = 0.093). The baseline characteristics of the participants stratified by serum hepcidin-25 tertiles are listed in Table 1. Patients in the lower hepcidin-25 tertile had lower levels of BMI, TSAT, and serum ferritin and higher total Kt/V compared to those in the higher tertile (*P* < 0.05).

**Table 1:** Baseline characteristics of individuals stratified by tertiles of baseline serum hepcidin-25 levels.

**Variable**	**Total (*n* = 513)**	**Serum hepcidin-25 (ng/mL)**			***P*-value^**a**^**
		**≤24.97(*n* = 171)**	**24.97–64.23 (*n* = 171)**	**≥64.23(*n* = 171)**	
Age (years)	46 ± 14	45 ± 14	46 ± 14	48 ± 14	0.179
Men (*n*, %)	309 (60)	93 (54)	108 (63)	108 (63)	0.160
Body mass index (kg/m^2^)	21.6 ± 3.1	20.9 ± 2.9	22.1 ± 3.1	21.8 ± 3.3	**0.001**
Diabetes (*n*, %)	122 (24)	32 (19)	44 (26)	46 (27)	0.157
History of CVD (*n*, %)	194 (38)	68 (40)	68 (40)	58 (34)	0.422
Charlson Comorbidity Index	3.4 ± 1.7	3.2 ± 1.7	3.4 ± 1.7	3.6 ± 1.7	0.091
Hemoglobin (g/L)	103 ± 21	104 ± 22	104 ± 20	102 ± 21	0.588
Albumin (g/L)	37.30 ± 5.03	37.17 ± 4.91	37.99 ± 4.80	36.74 ± 5.32	0.067
Hs-CRP (mg/L)	1.63 (0.58–4.81)	1.35 (0.55–4.31)	1.73 (0.62–4.47)	1.73 (0.66–7.22)	0.128
Corrected-calcium (mmol/L)	2.32 ± 0.18	2.32 ± 0.22	2.31 ± 0.21	2.32 ± 0.18	0.898
Phosphorus (mmol/L)	1.42 ± 0.43	1.42 ± 0.49	1.41 ± 0.40	1.42 ± 0.41	0.995
Uric acid (μmol/L)	427 ± 95	418 ± 92	431 ± 97	432 ± 95	0.284
Total cholesterol (mmol/L)	4.89 ± 1.35	4.90 ± 1.49	4.87 ± 1.33	4.90 ± 1.23	0.969
Triglyceride (mmol/L)	1.42 (0.99–1.98)	1.35 (0.92–1.87)	1.38 (1.01–2.04)	1.50 (1.03–1.90)	0.241
Total Kt/V	2.54 ± 0.69	2.65 ± 0.69	2.42 ± 0.62	2.53 ± 0.73	**0.009**
RRF (mL/min/1.73 m^2^)	3.55 (2.13–5.33)	3.51 (2.26–5.66)	3.79 (2.06–5.53)	3.55 (2.00–5.09)	0.565
Iron (μmol/L)	8.40 (5.20–12.90)	7.90 (4.00–12.80)	9.10 (6.20–12.60)	8.40 (5.43–13.30)	0.063
TIBC (μmol/L)	56.10 (46.75–67.35)	56.30 (49.00–69.20)	56.80 (46.70–67.60)	56.30 (46.90–65.40)	0.409
TSAT (%)	15 (9–24)	14 (7–23)	15 (10–25)	15 (11–24)	**0.014**
Ferritin (ng/mL)	132.76 (51.32–292.02)	63.47 (31.59–145.01)	161.50 (66.75–310.33)	222.32 (87.74–506.93)	** <0.001**
ESA users (*n*, %)	449 (89)	150 (91)	144 (85)	155 (92)	0.057
Iron users (*n*, %)	369 (72)	127 (75)	118 (70)	124 (73)	0.544

*Values expressed as mean ± SD, number (percent), or median (interquartile range)*.

a *P < 0.05 is considered statistically significant*.

*CVD, cardiovascular disease; Hs-CRP, high-sensitivity C-reactive protein; Kt/V urea, urea clearance (Kt) normalized to total body water (V); RRF, residual renal function; TIBC, total iron binding capacity; TSAT, transferrin saturation; ESA, erythropoiesis-stimulating agent*.

### Clinical Characteristics Associated With Serum Hepcidin-25 Levels

Univariate and multivariate models of clinical variables associated with baseline serum hepcidin-25 level are shown in Table 2. In the unadjusted model, serum hepcidin-25 level was positively correlated with BMI, uric acid, hs-CRP, serum iron, TIBC, serum ferritin, and TSAT levels and negatively correlated with female gender and total Kt/V (*P* < 0.05). The associations of serum hepcidin-25 level with BMI and serum ferritin were still significant even after adjustment for multiple confounders. However, age, CCI, hemoglobin, albumin, corrected-calcium, or phosphorus, total cholesterol, triglyceride and RRF were not associated with serum hepcidin-25 level.

**Table 2:** Clinical and laboratory factors associated with serum hepcidin-25 concentration ^#^.

**Variable**	**Univariate analysis**		**Multivariable analysis**	
	**Beta (95%CI)**	***P*-value**	**Beta (95%CI)**	***P*-value**
Age (years)	0.003 (−0.001, 0.007)	0.104	–	–
Gender (female vs. Male)	**−0.108 (−0.209**, **−0.007)**	**0.037**	–	–
Body mass index (kg/m^2^)	**0.028 (0.012, 0.044)**	** <0.001**	**0.016 (0.0001, 0.031)**	**0.044**
Charlson Comorbidity Index	0.029 (0.001, 0.058)	0.051	–	–
Hemoglobin (g/L)	−0.001 (−0.003, 0.002)	0.655	–	–
Albumin (g/L)	−0.002 (−0.012, 0.008)	0.750	–	–
Corrected-Calcium (mmol/L)	−0.034 (−0.279, 0.211)	0.786	–	–
Phosphorus (mmol/L)	−0.002 (−0.116, 0.113)	0.977	–	–
Total cholesterol (mmol/L)	−0.029 (−0.065, 0.008)	0.127	–	–
Triglyceride (mmol/L)	0.014 (−0.141, 0.287)	0.502	–	–
Uric acid (μmol/L)	**0.001 (0.0001, 0.001)**	**0.017**	–	–
Hs-CRP (mg/L)	**0.116 (0.028, 0.203)**	**0.009**	–	–
Iron (μmol/L)	**0.285 (0.125, 0.445)**	** <0.001**	–	–
TIBC (μmol/L)	**0.285 (0.125, 0.445)**	** <0.001**	–	–
Ferritin (ng/mL)	**0.448 (0.361, 0.535)**	** <0.001**	**0.435 (0.342, 0.527)**	** <0.001**
TSAT (%)	**0.345 (0.185, 0.505)**	** <0.001**	–	–
Total Kt/V	**−0.098 (−0.172**, **−0.025)**	**0.009**	–	–
RRF (mL/min/1.73 m^2^)	−0.064 (−0.208, 0.081)	0.387	–	–

# *Natural log-transformed serum hepcidin-25 was used as the dependent variable in univariable and a multivariable linear regression models to identify independent factors. Triglyceride, hs-CRP, iron, TIBC, ferritin, TSAT and RRF were also log-transformed. 95% CI, 95% confidence interval; Hs-CRP, high-sensitivity C-reactive protein; TIBC, total iron binding capacity; TSAT, transferrin saturation; Kt/V urea, urea clearance (Kt) normalized to total body water (V); RRF, residual renal function. Bold indicates significance at P < 0.05*.

### Serum Hepcidin-25 Levels and Mortality

**Figure 3:**
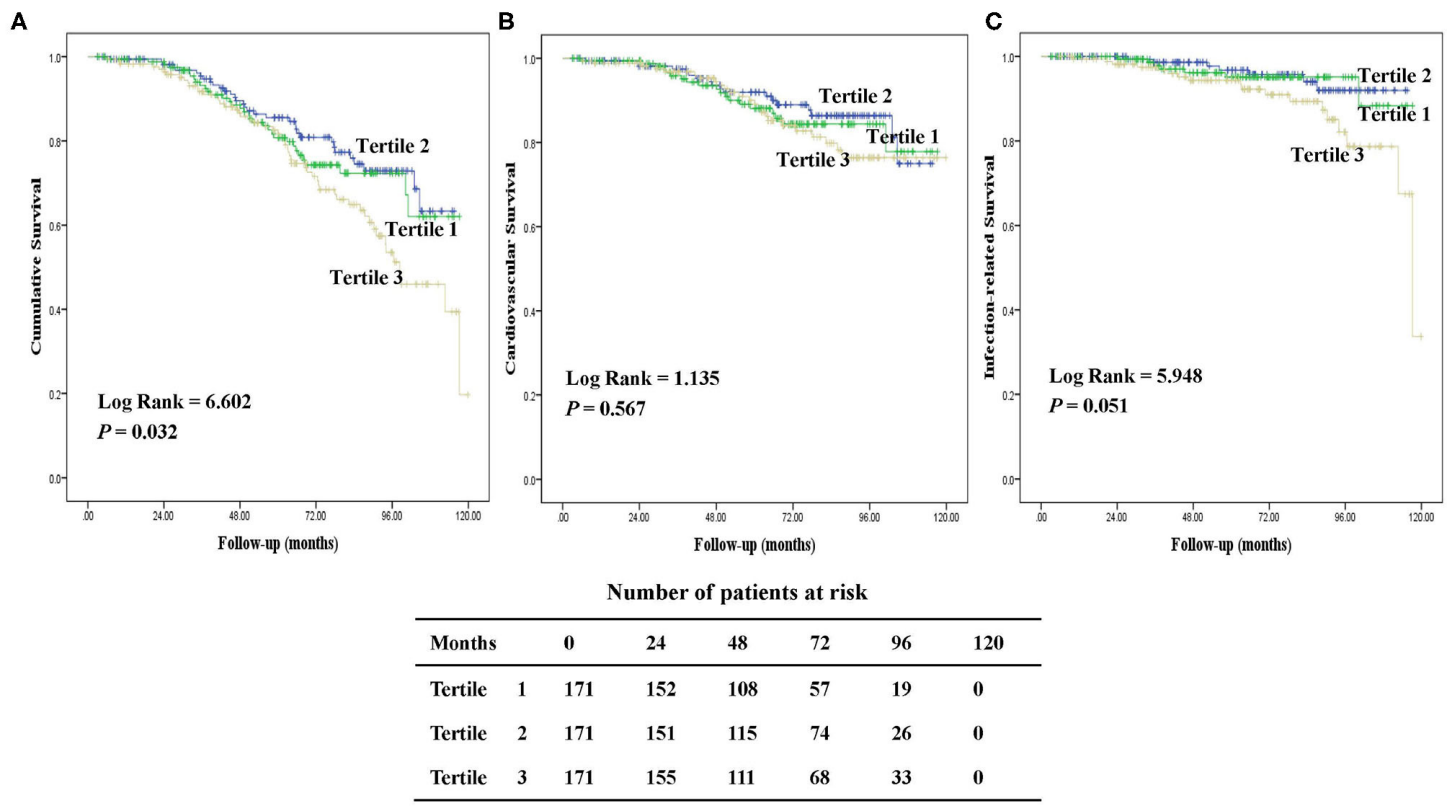
Kaplan-Meier survival curves for patients with tertiles of serum hepcidin-25. Cumulative mortality curves for all-cause mortality **(A)**, cardiovascular mortality **(B)**, and infection-related mortality **(C)**, according to the serum hepcidin-25 level at baseline.

During a median follow-up of 64.1 (IQR, 47.6–85.7) months, 122 (24%) patients died. The major causes of death were CV diseases (50%), followed by infection (26%) including peritonitis (47%) and other infection (53%). Figure 3 presents the Kaplan-Meier survival curves for all-cause, CV, and infection-related mortality by tertiles of baseline hepcidin-25 levels. Patients in the upper tertiles were likely to have worse all-cause survival rates among the groups (*P* = 0.032). However, there were no significant differences in CV and infection-related mortality across the groups.

**Figure 4:**
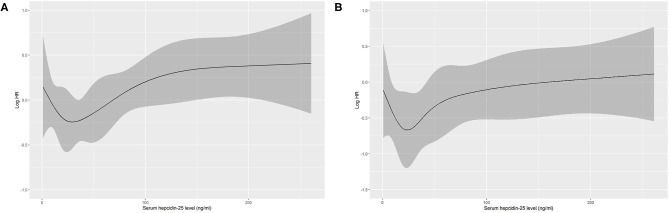
Association of serum hepcidin-25 levels with all-cause mortality in PD patients using restricted cubic spline. Baseline serum hepcidin-25 levels were modeled as a continuous variable, and the model was unadjusted **(A)** and multivariate **(B)** adjusted (adjusted for age, gender, body mass index, uric acid, corrected calcium, phosphorus, albumin, comorbidity score, hemoglobin, residual renal function, total Kt/V, high-sensitivity C-reactive protein level, serum ferritin and treatment of erythropoiesis-stimulating agents (ESAs) and/or iron). HR, hazard ratio.

Based on restricted cubic spline analysis, a trend toward *U*-shaped association was noted between serum hepcidin-25 and all-cause mortality in PD patients (Figure 4). We performed multivariate Cox and competing-risks regression analysis with the second tertile as a reference to assess the relationship between serum hepcidin-25 level and the risk of all-cause, CV, and infection-related mortality. As shown in Table 3, compared with the reference group, hepcidin-25 in the tertile 3 group was associated with a higher risk of all-cause [adjusted hazard ratio (aHR) 1.85, 95% confidence interval (95%CI), 1.14 to 3.00, *P* = 0.013] and infection-related mortality (adjusted subdistributional hazard ratio [aSHR], 2.61; 95%CI, 1.01 to 6.76, *P* = 0.049) after adjustment for potential confounders. However, no significant correlation was found between serum hepcidin-25 level and CV mortality.

**Table 3:** Associations of tertiles of serum hepcidin-25 with all-cause, cardiovascular and infection-related mortality.

	**No. of deaths (%)**	**Model 1**^****a****^		**Model 2**^****b****^		**Model 3**^****c****^	
**All-cause mortality**		**HR (95% CI)**	***P*****-value**	**HR (95% CI)**	***P*****-value**	**HR (95% CI)**	***P*****-value**
Tertile 1	34 (19.9)	1.20 (0.74–1.93)	0.463	1.17 (0.72–1.91)	0.526	1.31 (0.77–2.24)	0.315
Tertile 2	33 (19.3)	1.0		1.0		1.0	
Tertile 3	55 (32.2)	**1.71 (1.11**–**2.63)**	**0.015**	**1.83 (1.18**–**2.83)**	**0.007**	**1.85 (1.14**–**3.00)**	**0.013**
**Cardiovascular mortality**		**SHR (95% CI)**	***P*****-value**	**SHR (95% CI)**	***P*****-value**	**SHR (95% CI)**	***P*****-value**
Tertile 1	19 (11.1)	1.18 (0.62–2.25)	0.608	1.12 (0.59–2.12)	0.738	1.46 (0.71–2.98)	0.304
Tertile 2	18 (10.5)	1.0		1.0		1.0	
Tertile 3	24 (14.0)	1.32 (0.72–2.43)	0.369	1.24 (0.68–2.28)	0.482	1.33 (0.64–2.78)	0.443
**Infection-related mortality**		**SHR (95% CI)**	***P*****-value**	**SHR (95% CI)**	***P*****-value**	**SHR (95% CI)**	***P*****-value**
Tertile 1	7 (4.1)	1.11 (0.39–3.16)	0.847	1.24 (0.42–3.60)	0.699	0.80 (0.23–2.84)	0.735
Tertile 2	7 (4.1)	1.0		1.0		1.0	
Tertile 3	18 (10.5)	**2.42 (1.02**–**5.73)**	**0.045**	**2.61 (1.07**–**6.36)**	**0.034**	**2.61 (1.01**–**6.76)**	**0.049**

*HR, hazard ratio; SHR, Subdistribution hazard ratio; 95% CI, 95% confidence interval. Bold indicates significance at P < 0.05*.

a *Unadjusted model*.

b *Adjusted for age, gender, body mass index*.

c *Adjusted for model 2 covariates and uric acid, corrected calcium, phosphorus, albumin, comorbidity score, hemoglobin, residual renal function, total Kt/V, high-sensitivity C-reactive protein level, serum ferritin and treatment of erythropoiesis-stimulating agents (ESAs) and/or iron*.

## Discussion

In the current study, we demonstrated that BMI and serum ferritin were significant independent factors related to serum hepcidin-25. Moreover, this is the first study, to the best of our knowledge, to show that higher serum hepcidin-25 level is associated with increased risk of all-cause and infection-related mortality in PD patients.

The 25-amino acid peptide hormone hepcidin, which is encoded by hepcidin antimicrobial peptide, is responsible for systemic iron homeostasis through inhibiting the uptake of iron in the gut and preventing the release of stored iron from macrophages (21). Hepcidin is also important for innate immunity (22). In chronic inflammatory status, enhanced serum hepcidin synthesis has been correlated with increased interleukin-6 and CRP (23). Previous studies showed that dialysis patients had higher levels of serum hepcidin compared to general population, possibly due to impaired renal excretion, chronic inflammatory status, iron administration, ESA dose, and inadequate dialysis (24, 25). Our findings were consistent with previous studies indicating a positive correlation between serum hepcidin-25 level and ferritin. However, hepcidin-25 level was not significantly associated with serum iron, TAST, and the inflammation marker hs-CRP after adjustment of potential confounders. In addition, our results are in line with prior studies which showed that patients with higher BMI had relatively higher serum hepcidin levels (26, 27). This observation could be explained by that adipose tissue and higher levels of leptin in obesity individuals could increase the synthesis of hepcidin (28, 29).

Several researchers have evaluated the association between serum hepcidin level and mortality risk in CKD patients, but the results are controversial. Some studies have shown the association of hepcidin-25 with increased CV mortality risk in HD patients (15) or with all-cause mortality in diabetic CKD patients (30), while Eisenga et al. have not found a predictive role of hepcidin-25 in mortality among patients with kidney transplantation (31). Until now, there has no study to investigate the relationship between serum hepcidin level and mortality in PD patients. Our results showed that higher baseline hepcidin-25 level was associated with elevated all-cause mortality, suggesting that hepcidin-25 may be a useful assessment indicator to identify PD patients with higher mortality risk. There is a microsystemic chronic inflammatory state in PD patients (32), and the results of our present study reflect previous research (17) which has demonstrated a strong association between hepcidin-25 and inflammatory marker, such as CRP. One of the potential explanations of the relationship between higher hepcidin-25 levels and greater mortality is due to chronic inflammation. Interestingly, our results showed that the relationship between hepcidin-25 and all-cause mortality was non-linear, exhibiting approximate U-shaped association. This phenomenon may be attributed to the protective effects of hepcidin itself in a suitable range, such as anti-inflammatory effects (33) and regulating functional hematopoiesis (21). However, we did not find that lower hepcidin-25 levels were associated with patients' mortality. Some confounding factors may influence the effect of the hepcidin-25 level alone on the prognosis of PD patients.

High hepcidin level has been associated with increased susceptibility for several human and murine infectious diseases (34, 35). In the present study, our results showed that high serum hepcidin-25 was associated with increased risks of infection-related mortality in PD patients. A similar relationship was also found in patients with kidney transplants (36). However, the underlying mechanisms between serum hepcidin and infection-related mortality are not fully understood. Hepcidin acts as a host-defense mediator during early phase of infection (37), which leads to acute hypoferremia and suppresses the growth of microorganisms (38). It exhibits multifaceted mechanisms to fight with a variety of pathogens, including bacteria, viruses, fungi and parasites. For instance, hepcidin can bind to negatively charged cell membranes of microorganisms to penetrate and destroy them (39). Moreover, hepcidin is capable of enhancing the expression of immune-related genes (40). Conversely, hepcidin may be detrimental to cellular defense against certain intracellular infections through accelerating retention of iron in macrophages (12) and further losing its ability to kill pathogens, consistence with the clinical observation that patients with iron overload were associated with increased bacterial infection (41). Therefore, the clinical significance of hepcidin in predicting the risk of infection-related mortality may be complex, in part, depending on the pathogen and its niche. Although we have no data available regarding intracellular iron accumulation in PD patients, modulation of hepcidin induction might have a protective effect, including reducing infection burden and improving patients' survival. Nevertheless, further studies should be conducted to clarify our findings.

Hepcidin inhibition is a promising approach to treat CKD anemia and improve the quality of life. Great efforts have been made to produce hepcidin antagonists by pharmacological control of hepcidin expression or its activity. Hypoxia-inducible factor prolyl hydroxylase enzyme inhibitors, as a new class of agents for the therapy of anemia in CKD, increase endogenous erythropoietin and decrease serum hepcidin levels, leading to more effective internal iron metabolism without the need for excess iron administration in dialysis patients (42). Interestingly, insulin therapy and other diabetes drugs (such as SGLT2 inhibitors) have been suggested to suppress the production of hepcidin and modulate iron homeostasis, thereby increasing erythropoiesis and hematocrit (43, 44). Although correcting abnormally elevated hepcidin levels has beneficial effects on iron homeostasis, therapy with hepcidin antagonist should be administered cautiously in patients with acute infection (45, 46), due to the alteration of the host-pathogen competition for iron and promoting the infection processes.

There are several limitations to our study. Firstly, our study only examined the Chinese PD patients in a single-center, thus our findings may not be generalizable to the overall PD population. Secondly, hepcidin has significant intra-individual variability, which may depend on inflammatory fluctuations (47). However, the level of hepcidin-25 was only a single measurement, and we did not evaluate the relationship between longitudinal changes of hepcidin-25 and prognosis of PD patients. Moreover, some inflammatory markers such as interleukin-6 and tumor necrosis factor-α were not available in our data set, which may contribute to our results being partially biased by these unmeasured factors. Furthermore, a causal association between hepcidin-25 and hard outcomes cannot be established.

In conclusion, higher levels of serum hepcidin-25 are independently associated with increased risk of all-cause and infection-related mortality in PD patients. Further studies with a larger sample and multiple centers are needed to verify our findings and determine whether lowering serum hepcidin-25 is beneficial for improving the outcome of PD patients.

## Data Availability Statement

The raw data supporting the conclusions of this article will be made available by the authors, without undue reservation.

## Ethics Statement

The studies involving human participants were reviewed and approved by The Ethics Committee of The First Affiliated Hospital of Sun Yat-sen University. The patients/participants provided their written informed consent to participate in this study.

## Author Contributions

HM and ZZ designed the research. ZZ, DL, DF, and LF collected the data. ZZ, DL, and BL analyzed the data. ZZ and NL performed experiments. ZZ and DL wrote the paper. ZL, WC, and HM revised the manuscript. All authors contributed to the article and approved the submitted version.

## Conflict of Interest

The authors declare that the research was conducted in the absence of any commercial or financial relationships that could be construed as a potential conflict of interest.

## References

[1] Singh AK. Anemia of chronic kidney disease. Clin J Am Soc Nephrol. (2008) 3:3–6. 10.2215/CJN.05131107 18077779

[2] Cui Y Wu Q Zhou Y. Iron-refractory iron deficiency anemia: new molecular mechanisms. Kidney Int. (2009) 76:1137–41. 10.1038/ki.2009.357 19776721 PMC2869468

[3] Sankaran VG Weiss MJ. Anemia: progress in molecular mechanisms and therapies. Nat Med. (2015) 21:221–30. 10.1038/nm.3814 25742458 PMC4452951

[4] Santos EJF Hortegal EV Serra HO Lages JS Salgado-Filho N Dos Santos AM. Epoetin alfa resistance in hemodialysis patients with chronic kidney disease: a longitudinal study. Braz J Med Biol Res. (2018) 51:e7288. 10.1590/1414-431x20187288 29742267 PMC5972010

[5] Ogawa T Nitta K. Erythropoiesis-stimulating agent hyporesponsiveness in end-stage renal disease patients. Contrib Nephrol. (2015) 185:76–86. 10.1159/000380972 26023017

[6] Elliott J Mishler D Agarwal R. Hyporesponsiveness to erythropoietin: causes and management. Adv Chronic Kidney Dis. (2009) 16:94–100. 10.1053/j.ackd.2008.12.004 19233068

[7] Perlman RL Zhao J Fuller DS Bieber B Li Y Pisoni RL. International anemia prevalence and management in peritoneal dialysis patients. Perit Dial Int. (2019) 39:539–46. 10.3747/pdi.2018.00249 31582465

[8] Luo D Zhong Z Qiu Y Wang Y Li H Lin J. Abnormal iron status is associated with an increased risk of mortality in patients on peritoneal dialysis. Nutr Metab Cardiovasc Dis. (2021) 31:1148–55. 10.1016/j.numecd.2020.12.018 33618923

[9] Gafter-Gvili A Schechter A Rozen-Zvi B. Iron deficiency anemia in chronic kidney disease. Acta Haematol. (2019) 142:44–50. 10.1159/000496492 30970355

[10] Santos-Silva A Ribeiro S Reis F Belo L. Hepcidin in chronic kidney disease anemia. Vitam Horm. (2019) 110:243–64. 10.1016/bs.vh.2019.01.012 30798815

[11] Wojtaszek E Glogowski T Malyszko J. Iron and chronic kidney disease: still a challenge. Front Med (Lausanne). (2020) 7:565135. 10.3389/fmed.2020.565135 33392212 PMC7775475

[12] Michels K Nemeth E Ganz T Mehrad B. Hepcidin and host defense against infectious diseases. PLoS Pathog. (2015) 11:e1004998. 10.1371/journal.ppat.1004998 26291319 PMC4546197

[13] Ashby DR Gale DP Busbridge M Murphy KG Duncan ND Cairns TD. Plasma hepcidin levels are elevated but responsive to erythropoietin therapy in renal disease. Kidney Int. (2009) 75:976–81. 10.1038/ki.2009.21 19212416

[14] Zaritsky J Young B Wang HJ Westerman M Olbina G Nemeth E. Hepcidin–a potential novel biomarker for iron status in chronic kidney disease. Clin J Am Soc Nephrol. (2009) 4:1051–6. 10.2215/CJN.05931108 19406957 PMC2689881

[15] Yayar O Eser B Kilic H. Relation between high serum hepcidin-25 level and subclinical atherosclerosis and cardiovascular mortality in hemodialysis patients. Anatol J Cardiol. (2018) 19:117–22. 10.14744/AnatolJCardiol.2017.8019 29339674 PMC5864805

[16] Niikura T Maruyama Y Nakashima S Matsuo N Tanno Y Ohkido I. Hepcidin/ferritin ratios differ among non-dialyzed chronic kidney disease patients, and patients on hemodialysis and peritoneal dialysis. Ther Apher Dial. (2019) 23:341–6. 10.1111/1744-9987.12773 30411489

[17] Erdogan B Eser B Yayar O Ayli MD. The association between serum hepcidin-25 level and subclinical atherosclerosis in peritoneal dialysis patients. Turk Kardiyol Dern Ars. (2018) 46:121–8. 10.5543/tkda.2017.17666 29512612

[18] Ulu SM Yuksel S Altuntas A Kacar E Ahsen A Altug A. Associations between serum hepcidin level, FGF-21 level and oxidative stress with arterial stiffness in CAPD patients. Int Urol Nephrol. (2014) 46:2409–14. 10.1007/s11255-014-0753-7 24908281

[19] Charlson ME Pompei P Ales KL MacKenzie CR. A new method of classifying prognostic comorbidity in longitudinal studies: development and validation. J Chronic Dis. (1987) 40:373–83. 10.1016/0021-9681(87)90171-8 3558716

[20] Zhong Z Peng F Shi D Peng Y Li B Xiao M. Serum lipoprotein(a) and risk of mortality in patients on peritoneal dialysis. J Clin Lipidol. (2020) 14:252–9. 10.1016/j.jacl.2020.01.008 32081604

[21] van Swelm RPL Wetzels JFM Swinkels DW. The multifaceted role of iron in renal health and disease. Nat Rev Nephrol. (2020) 16:77–98. 10.1038/s41581-019-0197-5 31554933

[22] Armitage AE Eddowes LA Gileadi U Cole S Spottiswoode N Selvakumar TA. Hepcidin regulation by innate immune and infectious stimuli. Blood. (2011) 118:4129–39. 10.1182/blood-2011-04-351957 21873546

[23] Belo L Rocha S Valente MJ Coimbra S Catarino C Bronze-da-Rocha E. Hepcidin and diabetes are independently related with soluble transferrin receptor levels in chronic dialysis patients. Ren Fail. (2019) 41:662–72. 10.1080/0886022X.2019.1635893 31296086 PMC6691825

[24] Gluba-Brzozka A Franczyk B Olszewski R Rysz J. The influence of inflammation on anemia in CKD patients. Int J Mol Sci. (2020) 21:725. 10.3390/ijms21030725 31979104 PMC7036805

[25] Hamano H Ikeda Y Watanabe H Horinouchi Y Izawa-Ishizawa Y Imanishi M. The uremic toxin indoxyl sulfate interferes with iron metabolism by regulating hepcidin in chronic kidney disease. Nephrol Dial Transplant. (2018) 33:586–97. 10.1093/ndt/gfx252 28992067

[26] Vuppalanchi R Troutt JS Konrad RJ Ghabril M Saxena R Bell LN. Serum hepcidin levels are associated with obesity but not liver disease. Obesity (Silver Spring). (2014) 22:836–41. 10.1002/oby.20403 23512600 PMC3692602

[27] Moreno-Navarrete JM Moreno M Puig J Blasco G Ortega F Xifra G. Hepatic iron content is independently associated with serum hepcidin levels in subjects with obesity. Clin Nutr. (2017) 36:1434–9. 10.1016/j.clnu.2016.09.022 27745814

[28] Bekri S Gual P Anty R Luciani N Dahman M Ramesh B. Increased adipose tissue expression of hepcidin in severe obesity is independent from diabetes and NASH. Gastroenterology. (2006) 131:788–96. 10.1053/j.gastro.2006.07.007 16952548

[29] Chung B Matak P McKie AT Sharp P. Leptin increases the expression of the iron regulatory hormone hepcidin in HuH7 human hepatoma cells. J Nutr. (2007) 137:2366–70. 10.1093/jn/137.11.2366 17951471

[30] Wagner M Ashby DR Kurtz C Alam A Busbridge M Raff U. Hepcidin-25 in diabetic chronic kidney disease is predictive for mortality and progression to end stage renal disease. PLoS ONE. (2015) 10:e0123072. 10.1371/journal.pone.0123072 25894587 PMC4404250

[31] Eisenga MF Dullaart RP Berger SP Sloan JH de Vries AP Bakker SJ. Association of hepcidin-25 with survival after kidney transplantation. Eur J Clin Invest. (2016) 46:994–1001. 10.1111/eci.12682 27696386 PMC5132077

[32] Stenvinkel P Alvestrand A. Inflammation in end-stage renal disease: sources, consequences, and therapy. Semin Dial. (2002) 15:329–37. 10.1046/j.1525-139X.2002.00083.x 12358637

[33] Pagani A Nai A Corna G Bosurgi L Rovere-Querini P Camaschella C. Low hepcidin accounts for the proinflammatory status associated with iron deficiency. Blood. (2011) 118:736–46. 10.1182/blood-2011-02-337212 21628413

[34] Zhou C Chen Y Ji Y He X Xue D. Increased serum levels of hepcidin and ferritin are associated with severity of COVID-19. Med Sci Monit. (2020) 26:e926178. 10.12659/MSM.926178 32978363 PMC7526336

[35] Paradkar PN De Domenico I Durchfort N Zohn I Kaplan J Ward DM. Iron depletion limits intracellular bacterial growth in macrophages. Blood. (2008) 112:866–74. 10.1182/blood-2007-12-126854 18369153 PMC2481528

[36] Fernandez-Ruiz M Parra P Ruiz-Merlo T Lopez-Medrano F San Juan R Polanco N. Association between baseline serum hepcidin levels and infection in kidney transplant recipients: potential role for iron overload. Transpl Infect Dis. (2018) 20:e12807. 10.1111/tid.12807 29120522

[37] Drakesmith H Prentice AM. Hepcidin and the iron-infection axis. Science. (2012) 338:768–72. 10.1126/science.1224577 23139325

[38] Ruchala P Nemeth E. The pathophysiology and pharmacology of hepcidin. Trends Pharmacol Sci. (2014) 35:155–61. 10.1016/j.tips.2014.01.004 24552640 PMC3978192

[39] Zhang J Yu LP Li MF Sun L. Turbot (*Scophthalmus maximus*) hepcidin-1 and hepcidin-2 possess antimicrobial activity and promote resistance against bacterial and viral infection. Fish Shellfish Immunol. (2014) 38:127–34. 10.1016/j.fsi.2014.03.011 24647314

[40] Pan CY Peng KC Lin CH Chen JY. Transgenic expression of tilapia hepcidin 1-5 and shrimp chelonianin in zebrafish and their resistance to bacterial pathogens. Fish Shellfish Immunol. (2011) 31:275–85. 10.1016/j.fsi.2011.05.013 21642002

[41] Ribeiro S Belo L Reis F Santos-Silva A. Iron therapy in chronic kidney disease: recent changes, benefits and risks. Blood Rev. (2016) 30:65–72. 10.1016/j.blre.2015.07.006 26342303

[42] Chen N Hao C Liu BC Lin H Wang C Xing C. Roxadustat treatment for anemia in patients undergoing long-term dialysis. N Engl J Med. (2019) 381:1011–22. 10.1056/NEJMoa1901713 31340116

[43] Ghanim H Abuaysheh S Hejna J Green K Batra M Makdissi A. Dapagliflozin suppresses hepcidin and increases erythropoiesis. J Clin Endocrinol Metab. (2020) 105:dgaa057. 10.1210/clinem/dgaa057 32044999

[44] Vela D Sopi RB Mladenov M. Low hepcidin in type 2 diabetes mellitus: examining the molecular links and their clinical implications. Can J Diabetes. (2018) 42:179–87. 10.1016/j.jcjd.2017.04.007 28662967

[45] Malyszko J Malyszko JS Matuszkiewicz-Rowinska J. Hepcidin as a therapeutic target for anemia and inflammation associated with chronic kidney disease. Expert Opin Ther Targets. (2019) 23:407–21. 10.1080/14728222.2019.1599358 30907175

[46] Minchella PA Armitage AE Darboe B Jallow MW Drakesmith H Jaye A. Elevated hepcidin is part of a complex relation that links mortality with iron homeostasis and anemia in men and women with HIV infection. J Nutr. (2015) 145:1194–201. 10.3945/jn.114.203158 25904736 PMC4442111

[47] Ford BA Eby CS Scott MG Coyne DW. Intra-individual variability in serum hepcidin precludes its use as a marker of iron status in hemodialysis patients. Kidney Int. (2010) 78:769–73. 10.1038/ki.2010.254 20668427

